# Impact of serum phosphate changes on in-hospital mortality

**DOI:** 10.1186/s12882-020-02090-3

**Published:** 2020-10-07

**Authors:** Charat Thongprayoon, Wisit Cheungpasitporn, Panupong Hansrivijit, Sorkko Thirunavukkarasu, Api Chewcharat, Juan Medaura, Michael A. Mao, Kianoush B. Kashani

**Affiliations:** 1 Division of Nephrology and Hypertension, Department of Medicine, Mayo Clinic, 200 First St SW, Rochester, MN 55905 USA; 2 Division of Nephrology, Department of Internal Medicine, University of Mississippi Medical Center, Jackson, MS USA; 3 Department of Internal Medicine, University of Pittsburgh Medical Center Pinnacle, Harrisburg, PA USA; 4 Division of Nephrology and Hypertension, Mayo Clinic, Jacksonville, FL 32224 USA; 5 Division of Pulmonary and Critical Care Medicine, Department of Medicine, Mayo Clinic, Rochester, MN USA

**Keywords:** Phosphate, Electrolytes, Hypophosphatemia, Hyperphosphatemia, Mortality

## Abstract

**Background:**

Fluctuations in serum phosphate levels increased mortality in end-stage renal disease patients. However, the impacts of serum phosphate changes in hospitalized patients remain unclear. This study aimed to test the hypothesis that serum phosphate changes during hospitalization were associated with in-hospital mortality.

**Methods:**

We included all adult hospitalized patients from January 2009 to December 2013 that had at least two serum phosphate measurements during their hospitalization. We categorized in-hospital serum phosphate changes, defined as the absolute difference between the maximum and minimum serum phosphate, into 5 groups: 0–0.6, 0.7–1.3, 1.4–2.0, 2.1–2.7, ≥2.8 mg/dL. Using serum phosphate change group of 0–0.6 mg/dL as the reference group, the adjusted odds ratio of in-hospital mortality for various serum phosphate change groups was obtained by multivariable logistic regression analysis.

**Results:**

A total of 28,149 patients were studied. The in-hospital mortality in patients with serum phosphate changes of 0–0.6, 0.7–1.3, 1.4–2.0, 2.1–2.7, ≥2.8 mg/dL was 1.5, 2.0, 3.1, 4.4, and 10.7%, respectively (*p* < 0.001). When adjusted for confounding factors, larger serum phosphate changes were associated with progressively increased in-hospital mortality with odds ratios of 1.35 (95% 1.04–1.74) in 0.7–1.3 mg/dL, 1.98 (95% CI 1.53–2.55) in 1.4–2.0 mg/dL, 2.68 (95% CI 2.07–3.48) in 2.1–2.7 mg/dL, and 5.04 (95% CI 3.94–6.45) in ≥2.8 mg/dL compared to serum phosphate change group of 0–0.6 mg/dL. A similar result was noted when we further adjusted for either the admission or mean serum phosphate during hospitalization.

**Conclusion:**

Greater serum phosphate changes were progressively associated with increased in-hospital mortality.

## Background

Phosphate is an essential element for calcium-phosphate-parathyroid homeostasis, which plays an essential role in many cellular metabolisms [1–3]. Serum phosphate levels have been identified as strong predictors on clinical outcomes, including hospital mortality [4]. While hypophosphatemia is shown to be independently associated with increased mortality among critically ill patients, malnourished individuals, and patients with sepsis [5–7], hyperphosphatemia is associated with poor clinical outcomes including mortality in chronic kidney disease (CKD) [8, 9], hemodialysis [10, 11], acute coronary syndrome [12], and general patient population [13–19].

While previous studies have focused on the impact of serum phosphate disorders (hypophosphatemia and hyperphosphatemia) [5–7, 13–17], knowledge about the significance of changes in serum phosphate level and all-cause mortality remains scarce. Among patients on maintenance hemodialysis, a recent study demonstrated that high variability of serum phosphate was independently correlated with increased all-cause and cardiovascular mortality, while stable serum phosphate levels and low serum phosphate variability were associated with reduced patient mortality [10]. However, the impacts of serum phosphate changes in hospitalized patients remain unclear.

Therefore, we conducted this cohort study to assess the association between alteration of serum phosphate levels and in-hospital mortality in all hospitalized patients.

## Methods

### Study population

We included all hospitalized adult patients admitted to Mayo Clinic Rochester between January 1st, 2009, and December 31st, 2013, who had at least two serum phosphate measurements during hospital stay. The Mayo Clinic Institutional Review Board approved this study (IRB number 15–00024) and exempted the need for informed consent because this was a minimal risk study solely involving with chart review. For patients with multiple admissions during the study period, we included only the first hospital admission in analysis.

### Data collection

All serum phosphate values measured in hospital were reviewed. The predictor of interest was in-hospital serum phosphate changes, defined as the absolute difference between the in-hospital maximum and minimum serum phosphate. Serum phosphate changes were classified into 5 groups; 0–0.6, 0.7–1.3, 1.4–2.0, 2.1–2.7, ≥2.8 mg/dL. Our hospital used a photometric method to measure serum phosphate throughout the study period. A coefficient of variation using the photometric method was below 2% when serum phosphate was within the physiological range.

To further assess the effects of serum phosphate changes’ direction on mortality, the temporal relation between the maximum and minimum serum phosphate was evaluated. When the maximum serum phosphate preceded minimum serum phosphate, the decreasing trend of serum phosphate changes was assumed and the negative value of serum phosphate changes was assigned. When the minimum serum phosphate preceded maximum serum phosphate, the increasing trend of serum phosphate changes was assumed and the positive value of serum phosphate changes was assigned. Serum phosphate changes with the change’s direction were classified into 10 groups; ≤ − 2.8, − 2.7 to − 2.1, − 2.0 to − 1.4, − 1.3 to − 0.7, − 0.6 to 0.0, 0.1 to 0.6, 0.7 to 1.3, 1.4 to 2.0, 2.1 to 2.7, and ≥ 2.8 mg/dL.

Clinical characteristics included age, sex, race, principal diagnoses, comorbidities, estimated glomerular filtration rate (eGFR), acute kidney injury (AKI), the number of in-hospital serum phosphate measurements, and the length of hospital stay. The data collection of these clinical characteristics was described in our previous studies [20–22].

### Clinical outcomes

The primary outcome was in-hospital mortality, which was documented in the hospital database.

### Statistical analysis

Analysis of variance (ANOVA) and Chi-squared test were used respectively to compare continuous and categorical variables between serum phosphate change groups. Logistic regression was performed to report odds ratio (OR) with 95% confidence interval (CI) of the association between serum phosphate changes and in-hospital mortality, compared to serum phosphate change group of 0–0.6 mg/dL. Multivariable model was fitted to adjust for pre-specified variables. Model 1 was unadjusted; model 2 was adjusted for clinical characteristics. Model 3 was further adjusted for the admission serum phosphate, while model 4 was further adjusted for the mean in-hospital serum phosphate, in addition to all clinical characteristics in model 2. Pre-specified subgroup analysis based on AKI, CKD, and ESRD status was performed. Statistical significance achieved when 2-tailed *P* value < 0.05. All analyses were performed using JMP statistical software (Version 10; SAS Institute Inc).

## Results

### Clinical characteristics

A total of 28,149 patients were studied. 54% of enrolled patients were male. The mean age was 62 ± 17 years. The median number of in-hospital serum phosphate measurements was 4 (2–7), and length of hospital stay was 6 (4–11) days. The mean serum phosphate changes during hospital stay were 1.6 ± 1.4 mg/dL. Table 1 demonstrated the clinical characteristics based on serum phosphate change groups.

**Table 1:** Clinical characteristics

Variables	All	Changes in serum phosphate level during hospitalization (mg/dL)					
		0–0.6	0.7–1.3	1.4–2.0	2.1–2.7	≥2.8	*p*-value
N	28,149	6840	7459	5742	3713	4395	
Age (year)	62 ± 17	64 ± 17	63 ± 17	62 ± 17	61 ± 17	60 ± 17	< 0.001
Male sex	15,224 (54)	3690 (54)	4072 (55)	3091 (54)	1946 (52)	2425 (55)	0.12
Caucasian	25,650 (91)	6272 (92)	6808 (91)	5300 (92)	3362 (91)	3908 (89)	< 0.001
Principal diagnosis							< 0.001
- Cardiovascular	4650 (17)	1389 (20)	1230 (16)	817 (14)	479 (13)	735 (17)	
- Hematology/oncology	5864 (21)	1216 (18)	1543 (21)	1415 (25)	906 (24)	784 (18)	
- Infectious disease	1621 (6)	276 (4)	372 (5)	327 (6)	239 (6)	407 (9)	
- Endocrine/metabolic	1241 (4)	291 (4)	335 (5)	217 (4)	167 (4)	231 (5)	
- Respiratory	1423 (5)	350 (5)	375 (5)	295 (5)	196 (5)	207 (5)	
- Gastrointestinal	4274 (15)	994 (15)	1145 (15)	916 (16)	621 (17)	598 (14)	
- Genitourinary	1383 (5)	237 (3)	266 (4)	202 (4)	185 (5)	493 (11)	
- Injury and poisoning	4972 (18)	1200 (18)	1379 (18)	1061 (18)	634 (17)	698 (16)	
- Other	2721 (10)	887 (13)	814 (11)	492 (9)	286 (8)	242 (6)	
Charlson comorbidity score	2.3 ± 2.6	2.3 ± 2.6	2.3 ± 2.6	2.4 ± 2.7	2.3 ± 2.6	2.4 ± 2.5	0.24
Comorbidity							
- Coronary artery disease	5943 (21)	1611 (24)	1585 (21)	1156 (20)	702 (19)	889 (20)	< 0.001
- Congestive heart failure	2299 (8)	573 (8)	600 (8)	433 (8)	259 (7)	434 (10)	< 0.001
- Peripheral vascular disease	1211 (4)	285 (4)	342 (5)	258 (4)	140 (4)	186 (4)	0.30
- Stroke	2211 (8)	619 (9)	592 (8)	418 (7)	250 (7)	332 (8)	< 0.001
- Diabetes mellitus	6743 (24)	1618 (24)	1748 (23)	1318 (23)	851 (23)	1208 (27)	< 0.001
- COPD	2986 (11)	700 (10)	825 (11)	578 (10)	403 (11)	480 (11)	0.28
- Cirrhosis	1076 (4)	234 (3)	267 (4)	186 (3)	148 (4)	241 (5)	< 0.001
eGFR (ml/min/1.73 m^2^)	70 ± 34	74 ± 31	74 ± 32	73 ± 33	70 ± 35	53 ± 39	< 0.001
Acute kidney injury	11,692 (42)	2167 (32)	2627 (35)	2217 (39)	1642 (44)	3039 (69)	< 0.001
Number of serum phosphate measurement during hospitalization	4 (2–7)	2 (2–3)	3 (2–4)	4 (3–7)	6 (4–10)	10 (6–19)	< 0.001
Length of hospital stay (day)	6 (4–11)	4 (3–7)	5 (3–8)	7 (5–11)	9 (5–15)	12 (6–24)	< 0.001
Admission serum phosphate (mg/dL)	3.8 ± 1.3	3.5 ± 0.8	3.6 ± 0.9	3.7 ± 1.0	4.0 ± 1.2	4.9 ± 2.0	< 0.001
Mean serum phosphate (mg/dL)	3.5 ± 0.9	3.5 ± 0.8	3.4 ± 0.8	3.4 ± 0.8	3.5 ± 0.8	4.0 ± 1.1	< 0.001
Lowest serum phosphate (mg/dL)	2.8 ± 0.9	3.3 ± 0.8	2.9 ± 0.8	2.6 ± 0.8	2.4 ± 0.8	2.2 ± 0.9	< 0.001
Highest serum phosphate (mg/dL)	4.4 ± 1.3	3.7 ± 0.8	3.9 ± 0.8	4.3 ± 0.8	4.7 ± 0.9	6.3 ± 1.8	< 0.001

Continuous data are presented as mean ± SD or median (IQR); categorical data are presented as count (%)

Convert serum phosphate from mg/dL to mmol/L by multiplying by 0.32

### Serum phosphate changes and in-hospital mortality

Among 28,149 patients, 1060 (3.8%) died in hospital. The in-hospital mortality in patients with serum phosphate changes of 0–0.6, 0.7–1.3, 1.4–2.0, 2.1–2.7, ≥2.8 mg/dL was 1.5, 2.0, 3.1, 4.4, and 10.7%, respectively (*p* < 0.001) (Table 2). When adjusting for confounding factors in model 2, increased serum phosphate changes were progressively associated with increased in-hospital mortality with adjusted odds ratios of 1.35 (95% 1.04–1.74) in serum phosphate changes of 0.7–1.3, 1.98 (95% CI 1.53–2.55) in 1.4–2.0, 2.68 (95% CI 2.07–3.48) in 2.1–2.7, and 5.04 (95% CI 3.94–6.45) in ≥2.8 mg/dL, respectively, compared to serum phosphate change group of 0–0.6 mg/dL. When serum phosphate changes were analyzed as a continuous variable, an increase in serum phosphate changes by 1 mg/dL was associated with increased in-hospital mortality with an adjusted odds ratio of 1.33 (95% CI 1.28–1.38). A similar result was noted when we further adjusted for either the admission (model 3) and mean serum phosphate during hospitalization (model 4). The association of serum phosphate changes and in-hospital mortality did not differ by the duration of serum phosphate changes (p-interaction = 0.49).

**Table 2:** The association between serum phosphate changes and in-hospital mortality

Outcome	Changes in serum phosphate level during hospitalization (mg/dL)				
	0–0.6	0.7–1.3	1.4–2.0	2.1–2.7	≥2.8
In-hospital mortality	101 (1.5)	152 (2.0)	176 (3.1)	162 (4.4)	469 (10.7)
Mortality, OR (95% CI)					
- Model 1: unadjusted	1 (ref)	1.39 (1.08–1.79)	2.11 (1.65–2.70)	3.04 (2.37–3.92)	7.97 (6.41–9.92)
- Model 2^a^	1 (ref)	1.35 (1.04–1.74)	1.98 (1.53–2.55)	2.68 (2.07–3.48)	5.04 (3.94–6.45)
- Model 3: model 2 and admission serum phosphate	1 (ref)	1.39 (1.05–1.83)	1.99 (1.52–2.61)	2.75 (2.08–3.63)	4.79 (3.66–6.29)
- Model 4: model 2 and mean serum phosphate	1 (ref)	1.40 (1.08–1.82)	2.05 (1.59–2.65)	2.73 (2.10–3.55)	4.29 (3.33–5.52)

^a^Adjusted for age, sex, race, principal diagnosis, Charlson comorbidities score, history of coronary artery disease, congestive heart failure, peripheral artery disease, stroke, diabetes mellitus, chronic obstructive pulmonary disease, cirrhosis, eGFR, AKI, the number of serum phosphate measurement during hospitalization, and length of stay

Convert serum phosphate from mg/dL to mmol/L by multiplying by 0.32

A sensitivity analysis was performed in 23,049 patients with available serum calcium during hospitalization to further adjust association for mean serum calcium. In the sensitivity analysis, increased serum phosphate changes remained significantly associated with increased in-hospital mortality with adjusted odds ratios of 1.32 (95% 1.01–1.75) in serum phosphate changes of 0.7–1.3, 1.82 (95% CI 1.38–2.39) in 1.4–2.0, 2.35 (95% CI 1.77–3.11) in 2.1–2.7, and 3.38 (95% CI 2.58–4.43) in ≥2.8 mg/dL, respectively, compared to serum phosphate change group of 0–0.6 mg/dL.

### Direction of serum phosphate changes and in-hospital mortality

**Fig. 1:**
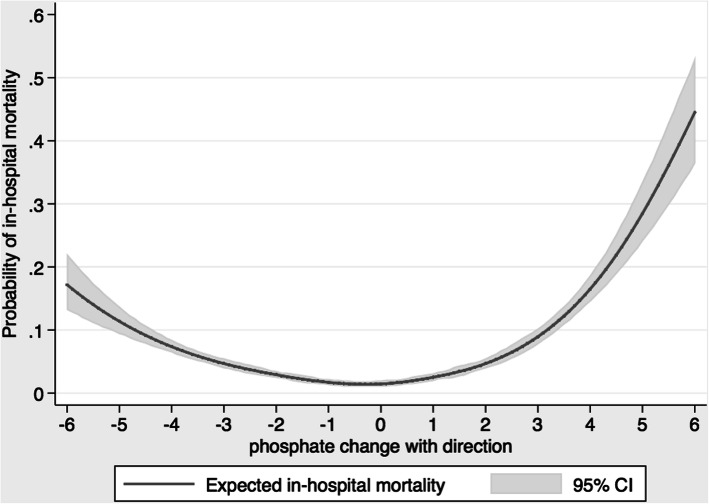
restricted cubic spline showed U-shaped association between direction of serum phosphate changes and in-hospital mortality

The lowest in-hospital mortality was observed in patients with serum phosphate change of − 0.6 to 0.0 mg/dL. A U-shaped distribution demonstrated higher in-hospital mortality associated with both decreasing and increasing trend of serum phosphate changes during hospitalization (Table 3 and Fig. 1). For the negative trend of serum phosphate, serum phosphate changes of ≤ − 2.8, − 2.7 to − 2.1, − 2.0 to − 1.4 mg/dL were significantly associated with increased in-hospital mortality. For the positive trend of serum phosphate, serum phosphate changes of 0.7 to 1.3, 1.4 to 2.0, 2.1 to 2.7, and ≥ 2.8 mg/dL were significantly associated with increased in-hospital mortality. Of note, the risk associated with markedly increasing trend of serum phosphate changes exceeded the risk related to markedly decreasing trend of serum phosphate changes.

**Table 3:** The association between direction of serum phosphate changes and in-hospital mortality

Phosphate change (mg/dL)	N	In-hospital mortality	Model 1	Model2	Model 3	Model 4
≤ −2.8	2941	230 (7.8)	6.74 (4.91–9.26)	4.32 (3.07–6.08)	2.94 (2.02–4.28)	3.70 (2.62–5.23)
−2.7 to − 2.1	2281	76 (3.3)	2.74 (1.90–3.96)	2.42 (1.66–3.53)	2.20 (1.48–3.26)	2.48 (1.70–3.62)
−2.0 to −1.4	3519	90 (2.6)	2.09 (1.46–2.98)	2.00 (1.39–2.87)	1.79 (1.22–2.63)	2.05 (1.43–2.96)
−1.3 to −0.7	4358	76 (1.7)	1.41 (0.98–2.03)	1.39 (0.96–2.01)	1.27 (0.85–1.88)	1.43 (0.99–2.08)
−0.6 to 0	3782	47 (1.2)	1 (ref)	1 (ref)	1 (ref)	1 (ref)
0.1 to 0.6	3058	54 (1.8)	1.43 (0.96–2.12)	1.50 (1.01–2.24)	1.47 (0.95–2.27)	1.48 (0.99–2.21)
0.7 to 1.3	3101	76 (2.5)	2.00 (1.38–2.88)	2.02 (1.39–2.93)	2.39 (1.61–3.55)	2.08 (1.43–3.02)
1.4 to 2.0	2223	86 (3.9)	3.20 (2.23–4.58)	3.13 (2.17–4.52)	3.57 (2.41–5.29)	3.24 (2.24–4.70)
2.1 to 2.7	1432	86 (6.0)	5.08 (3.54–7.28)	4.87 (3.35–7.08)	5.48 (3.67–8.17)	4.81 (3.30–7.02)
≥ 2.8	1454	239 (16.4)	15.63 (11–36-21.51)	10.26 (7.26–14.49)	11.22 (7.76–16.24)	8.56 (6.03–12.15)

Model 1: unadjusted

Model 2: Adjusted for age, sex, race, principal diagnosis, Charlson comorbidities score, history of coronary artery disease, congestive heart failure, peripheral artery disease, stroke, diabetes mellitus, chronic obstructive pulmonary disease, cirrhosis, eGFR, AKI, the number of serum phosphate measurement during hospitalization, and length of stay

Model 3: model 2 and the admission phosphate

Model 4: model 2 and mean serum phosphate during hospitalization

Convert serum phosphate from mg/dL to mmol/L by multiplying by 0.32

### Subgroup analysis based on AKI occurrence during hospitalization

11,692 (42%) patients developed AKI in hospital. In AKI and non-AKI patients, increased serum phosphate changes, especially when serum phosphate change ≥1.4 mg/dL, remained significantly associated with increased in-hospital mortality (Table S1). The association of serum phosphate changes with in-hospital mortality did not substantially differ in AKI and non-AKI patients (*p*-value for interaction = 0.60).

### Subgroup analysis based on CKD and ESRD status

In CKD patients, increased serum phosphate change of ≥1.4 mg/dL was significantly associated with increased in-hospital mortality. In non-CKD patients, increased serum phosphate change of ≥0.7 mg/dL was significantly associated with increased in-hospital mortality (Table S2). The association of serum phosphate changes with in-hospital mortality did not substantially differed between CKD and non-CKD patients (*p*-value for interaction = 0.68).

In ESRD patients, only increased serum phosphate change of ≥2.8 mg/dL was significantly associated with increased in-hospital mortality. In non-ESRD patients, increased serum phosphate change of ≥0.7 mg/dL was progressively associated with increased in-hospital mortality (Table S3). There was interaction between serum phosphate changes and ESRD status on in-hospital mortality (p-value for interaction = 0.02).

## Discussion

In this study, we report that changes in serum phosphate levels were significantly associated with mortality among all hospitalized patients. Greater serum phosphate changes were progressively associated with increased in-hospital mortality, while patients with stable serum phosphate had lower in-hospital mortality. There was a U-shaped distribution for in-hospital mortality associated with both decreasing and increasing trend of serum phosphate changes during hospitalization.

Recently, Zhu et al. conducted a retrospective cohort of 502 ESRD patients and found that low variability of serum phosphate levels in maintenance hemodialysis patients was significantly associated with reduced all-cause and cardiovascular disease mortality [10]. However, the populations enrolled in their study were limited to hemodialysis patients. Our study showed that regardless of ESRD status among all hospitalized patients, individuals with stable serum phosphate levels had lower hospital mortality in comparison with those with an absolute change of serum phosphate level ≥ 2.8 mg/dL.

It is known that an increase in serum phosphate is commonly seen in AKI, and AKI is frequent during hospitalization. Also, AKI is associated with higher mortality per se [23, 24]. We found that considerable serum phosphate changes are related to increased in-hospital mortality independent of AKI. Previous studies have shown an association between elevated serum phosphate levels and higher cardiovascular events, even in non-CKD patients [14, 25, 26]. An abrupt increase in serum phosphate levels may increase the risk of calcium phosphate precipitation, leading to hypocalcemia [27]. Life-threatening arrhythmias and cardiac arrest associated with hypocalcemia following the abrupt increase in serum phosphate have been reported [27–29], especially in patients with coronary artery disease [27]. Besides, it has been proposed that an increase in serum phosphate can result in the production of reactive oxygen species, decreased nitric oxide production via inhibitory phosphorylation of endothelial nitric oxide synthase [30], and inhibition of endothelium-dependent vasodilation. A similar finding reported in human subjects; that is, flow-mediated dilation of the brachial artery was significantly reduced following oral phosphate loads [30].

Reduction in serum phosphate level during hospitalization can occur among patients who receive insulin for treatment of diabetic ketoacidosis, sepsis, alcoholism, urinary phosphate-wasting syndromes, malnutrition and refeeding syndrome, and postoperative patients [14, 25, 26, 31–36]. A severe and abrupt decrease in serum phosphate levels can result in rhabdomyolysis, respiratory failure, lethargy, and confusion [1]. A reduction in serum phosphate level is also commonly found in severe AKI requiring renal replacement therapy [37]. Hypophosphatemia during renal replacement therapy could result in myocardial dysfunction, and prolonged respiratory failure [38, 39]. Among patients on chronic maintenance hemodialysis, higher intra-dialysis serum phosphate reduction ratio is independently associated with increased all-cause and cardiovascular mortality [40].

There are several limitations to our study. Given a single-center retrospective cohort study design, a causal association between serum phosphate changes and mortality rates could not be confirmed. It is well known that multiple factors can affect serum phosphate levels. Serum phosphate fluctuation could be a surrogate marker of illness severity, comorbidity burden, length of hospital stay, number of blood tests, or renal function, which could be independently associated with higher mortality rates [9, 35]. We adjusted the association for these potential confounders, and also performed additional subgroup analysis based on in-hospital AKI occurrence to mitigate these potential biases. However, the association between serum phosphate changes and mortality might remain confounded by residual or unmeasured confounders. This study retrieved the pertinent data from the hospital electronic database. However, some important clinical information such as hypophosphatemia or hyperphosphatemia treatments, including the use of intravenous or oral phosphate supplements, phosphate binders, serum phosphate-altering medications, urine phosphate excretion, and other biochemical data of mineral metabolism (e.g., vitamin D, and parathyroid hormone), was not available in our database and, therefore, we were not able to account for them in the analysis. Given the nature of this observational study, we could only capture serum phosphate that was measured in the clinical practice. Therefore, we might have missed the actual highest and lowest serum phosphate levels when serum phosphate was not measured. Furthermore, serum phosphate can have a significant biological variation. Thus, it is difficult to define the extent of serum phosphorus alteration as a pathological change. The biological change can incorporate a proportion of patients into the non-referent group, but this will bias the result toward the null hypothesis. The study population was predominantly white, potentially limiting generalizability of the study. Lastly, only the impact of serum phosphate changes’ magnitude and direction on mortality were addressed in our investigation, whereas other aspects of serum phosphate changes such as serum phosphate changes’ variability and acuity were not studied. These aspects are also important to the understanding of the relationship between serum phosphate changes with patient outcomes.

## Conclusion

We have shown that the serum phosphate alteration ≥0.7 mg/dL during hospitalization is associated with increased in-hospital mortality. The magnitude of association progressively increases with the degree of serum phosphate changes. Our study also confirms consistent associations regardless of AKI, CKD, and ESRD status. Future studies are required to evaluate if early identification of high mortality risk patients by including the degree of serum phosphate changes in risk prediction models allows clinicians to closely monitor these patients, initiate effective preventive and therapeutic strategies to minimize serum phosphate fluctuation, and ultimately reduce mortality among hospitalized patients.

## Supplementary information

**Additional file 1: Table S1.** Subgroup analysis based on in-hospital acute kidney injury status. **Table S2.** Subgroup analysis based on chronic kidney disease status. **Table S3.** Subgroup analysis based on end-stage kidney renal disease status.

## Data Availability

A limited de-identified dataset of the current study would be available per request. For the de-identified dataset, administrative permission is not required based on the institutional policies.

## Abbreviations

AKI: Acute kidney injury
CI: Confidence interval
CKD: Chronic kidney disease
eGFR: Estimated glomerular filtration rate
ESRD: End-stage renal disease
mg/dL: Milligram per deciliter
OR: Odds ratio
SD: Standard deviation
