# Supplementary material for: Impact of serum phosphate changes on in-hospital mortality
Source: BMC Nephrol. 2020 Oct 7;21:427. doi: 10.1186/s12882-020-02090-3 (PMC7542949; doi:10.1186/s12882-020-02090-3)
Supplement: Supplementary file 1 — Additional file 1: Table S1. Subgroup analysis based on in-hospital acute kidney injury status. Table S2. Subgroup analysis based on chronic kidney disease status. Table S3. Subgroup analysis based on end-stage kidney renal disease status. [file 12882_2020_2090_MOESM1_ESM.docx]

Table S1 subgroup analysis based on in-hospital acute kidney injury status

| Outcome | Changes in serum phosphate level during hospitalization (mg/dL) | | | | |
| --- | --- | --- | --- | --- | --- |
|  | 0-0.6 | 0.7-1.3 | 1.4-2.0 | 2.1-2.7 | ≥2.8 |
| AKI during hospitalization (n=11692) | | | | | |
| N | 2167 | 2627 | 2217 | 1642 | 3039 |
| Hospital mortality | 63 (2.9) | 97 (3.7) | 116 (5.2) | 112 (6.8) | 419 (13.8) |
| Mortality, OR (95% CI) |  |  |  |  |  |
| - Model 1: unadjusted | 1 (ref) | 1.28 (0.93-1.77) | 1.84 (1.35-2.52) | 2.44 (1.78-3.35) | 5.34 (4.07-7.00) |
| - Model 2# | 1 (ref) | 1.29 (0.93-1.80) | 1.87 (1.36-2.58) | 2.47 (1.79-3.42) | 4.92 (3.66-6.62) |
| - Model 3: model 2 and admission serum phosphate | 1 (ref) | 1.26 (0.89-1.80) | 1.83 (1.30-2.57) | 2.45 (1.74-3.47) | 4.40 (3.18-6.09) |
| - Model 4: model 2 and mean serum phosphate | 1 (ref) | 1.35 (0.97-1.87) | 1.93 (1.39-2.66) | 2.45 (1.76-3.41) | 3.92 (2.90-5.32) |
| No AKI during hospitalization (n=16457) | | | | | |
| N | 4673 | 4832 | 3525 | 2071 | 1356 |
| Hospital mortality | 38 (0.8) | 55 (1.1) | 60 (1.7) | 50 (2.4) | 50 (3.7) |
| Mortality, OR (95% CI) |  |  |  |  |  |
| - Model 1: unadjusted | 1 (ref) | 1.40 (0.93-2.13) | 2.11 (1.40-3.18) | 3.02 (1.97-4.62) | 4.67 (3.05-7.15) |
| - Model 2# | 1 (ref) | 1.41 (0.92-2.14) | 2.01 (1.32-3.06) | 2.88 (1.83-4.51) | 4.32 (2.64-7.07) |
| - Model 3: model 2 and admission serum phosphate | 1 (ref) | 1.59 (1.01-2.50) | 2.20 (1.39-3.46) | 3.29 (2.02-5.34) | 5.25 (3.10-8.92) |
| - Model 4: model 2 and mean serum phosphate | 1 (ref) | 1.37 (0.90-2.09) | 1.95 (1.28-2.97) | 2.80 (1.79-4.40) | 4.34 (2.65-7.12) |

#Adjusted for age, sex, race, principal diagnosis, Charlson comorbidities score, history of coronary artery disease, congestive heart failure, peripheral artery disease, stroke, diabetes mellitus, chronic obstructive pulmonary disease, cirrhosis, eGFR, the number of serum phosphate measurement during hospitalization, and length of stay

Table S2 subgroup analysis based on chronic kidney disease

| Outcome | Changes in serum phosphate level during hospitalization (mg/dL) | | | | |
| --- | --- | --- | --- | --- | --- |
|  | 0-0.6 | 0.7-1.3 | 1.4-2.0 | 2.1-2.7 | ≥2.8 |
| Chronic kidney disease (n=10544) | | | | | |
| N | 2189 | 2415 | 1938 | 1420 | 2582 |
| Hospital mortality | 52 (2.4) | 75 (3.1) | 91 (4.7) | 75 (5.3) | 293 (11.4) |
| Mortality, OR (95% CI) |  |  |  |  |  |
| - Model 1: unadjusted | 1 (ref) | 1.32 (0.92-1.89) | 2.02 (1.43-2.86) | 2.29 (1.60-3.29) | 5.26 (3.89-7.11) |
| - Model 2# | 1 (ref) | 1.27 (0.88-1.83) | 1.96 (1.37-2.79) | 2.24 (1.54-3.26) | 4.48 (3.19-6.29) |
| - Model 3: model 2 and admission serum phosphate | 1 (ref) | 1.23 (0.83-1.81) | 1.91 (1.31-2.78) | 2.27 (1.53-3.36) | 3.60 (2.48-5.21) |
| - Model 4: model 2 and mean serum phosphate | 1 (ref) | 1.33 (0.92-1.93) | 2.02 (1.41-2.91) | 2.25 (1.54-3.29) | 3.71 (2.62-5.25) |
| No chronic kidney disease (n=17605) | | | | | |
| N | 4651 | 5044 | 3804 | 2293 | 1813 |
| Hospital mortality | 49 (1.1) | 77 (1.5) | 85 (2.2) | 87 (3.8) | 176 (9.7) |
| Mortality, OR (95% CI) |  |  |  |  |  |
| - Model 1: unadjusted | 1 (ref) | 1.46 (1.02-2.09) | 2.15 (1.51-3.06) | 3.70 (2.60-5.28) | 10.10 (7.32-1.93) |
| - Model 2# | 1 (ref) | 1.43 (1.00-2.07) | 1.95 (1.36-2.81) | 3.16 (2.18-4.56) | 5.50 (3.80-7.97) |
| - Model 3: model 2 and admission serum phosphate | 1 (ref) | 1.56 (1.05-2.32) | 2.06 (1.38-3.05) | 3.44 (2.30-5.15) | 6.48 (4.33-9.70) |
| - Model 4: model 2 and mean serum phosphate | 1 (ref) | 1.46 (1.02-2.11) | 2.01 (1.40-2.89) | 3.25 (2.24-4.70) | 5.31 (3.66-7.70) |

#Adjusted for age, sex, race, principal diagnosis, Charlson comorbidities score, history of coronary artery disease, congestive heart failure, peripheral artery disease, stroke, diabetes mellitus, chronic obstructive pulmonary disease, cirrhosis, eGFR, AKI, the number of serum phosphate measurement during hospitalization, and length of stay

Table S3 subgroup analysis based on end-stage kidney renal disease status

| Outcome | Changes in serum phosphate level during hospitalization (mg/dL) | | | | |
| --- | --- | --- | --- | --- | --- |
|  | 0-0.6 | 0.7-1.3 | 1.4-2.0 | 2.1-2.7 | ≥2.8 |
| End-stage renal disease (n=2,079) | | | | | |
| N | 232 | 326 | 337 | 324 | 860 |
| Hospital mortality | 5 (2.2) | 9 (2.8) | 10 (3.0) | 8 (2.5) | 52 (6.1) |
| Mortality, OR (95% CI) |  |  |  |  |  |
| - Model 1: unadjusted | 1 (ref) | 1.19 (0.43-3.90) | 1.39 (0.47-4.12) | 1.15 (0.37-3.56) | 2.92 (1.15-7.40) |
| - Model 2# | 1 (ref) | 1.13 (0.36-3.55) | 1.15 (0.37-3.58) | 1.15 (0.35-3.77) | 2.92 (1.07-8.02) |
| - Model 3: model 2 and admission serum phosphate | 1 (ref) | 1.31 (0.37-4.58) | 1.26 (0.36-4.39) | 1.44 (0.40-5.11) | 3.40 (1.11-10.43) |
| - Model 4: model 2 and mean serum phosphate | 1 (ref) | 1.24 (0.39-3.90) | 1.21 (0.38-3.82) | 1.24 (0.38-4.09) | 2.96 (1.07-8.16) |
| No end-stage renal disease (n=26,070) | | | | | |
| N | 6608 | 7133 | 5405 | 3389 | 3535 |
| Hospital mortality | 96 (1.5) | 143 (2.0) | 166 (3.1) | 154 (4.5) | 417 (11.8) |
| Mortality, OR (95% CI) |  |  |  |  |  |
| - Model 1: unadjusted | 1 (ref) | 1.39 (1.07-1.80) | 2.15 (1.67-2.77) | 3.23 (2.49-4.18) | 9.07 (7.24-11.37) |
| - Model 2# | 1 (ref) | 1.36 (1.05-1.78) | 2.06 (1.59-2.67) | 2.92 (2.23-3.82) | 5.57 (4.31-7.19) |
| - Model 3: model 2 and admission serum phosphate | 1 (ref) | 1.41 (1.06-1.87) | 2.07 (1.56-2.74) | - 1. 2.24-3.98) | 5.26 (3.96-6.97) |
| - Model 4: model 2 and mean serum phosphate | 1 (ref) | 1.42 (1.09-1.86) | 2.14 (1.64-2.78) | 3.01 (2.30-3.95) | 4.65 (3.58-6.05) |

#Adjusted for age, sex, race, principal diagnosis, Charlson comorbidities score, history of coronary artery disease, congestive heart failure, peripheral artery disease, stroke, diabetes mellitus, chronic obstructive pulmonary disease, cirrhosis, eGFR, AKI, the number of serum phosphate measurement during hospitalization, and length of stay
